# A new paradigm in sweat based wearable diagnostics biosensors using Room Temperature Ionic Liquids (RTILs)

**DOI:** 10.1038/s41598-017-02133-0

**Published:** 2017-05-16

**Authors:** Rujuta D. Munje, Sriram Muthukumar, Badrinath Jagannath, Shalini Prasad

**Affiliations:** 1 Department of Bioengineering, The University of Texas at Dallas, Richardson, Texas 75080 USA; 2 EnLiSense LLC, 1813 Audubon Pond Way, Allen, Texas 75013 USA

## Abstract

Successful commercialization of wearable diagnostic sensors necessitates stability in detection of analytes over prolonged and continuous exposure to sweat. Challenges are primarily in ensuring target disease specific small analytes (i.e. metabolites, proteins, etc.) stability in complex sweat buffer with varying pH levels and composition over time. We present a facile approach to address these challenges using RTILs with antibody functionalized sensors on nanoporous, flexible polymer membranes. Temporal studies were performed using both infrared spectroscopic, dynamic light scattering, and impedimetric spectroscopy to demonstrate stability in detection of analytes, Interleukin-6 (IL-6) and Cortisol, from human sweat in RTILs. Temporal stability in sensor performance was performed as follows: (a) detection of target analytes after 0, 24, 48, 96, and 168 hours post-antibody sensor functionalization; and (b) continuous detection of target analytes post-antibody sensor functionalization. Limit of detection of IL-6 in human sweat was 0.2 pg/mL for 0–24 hours and 2 pg/mL for 24–48 hours post-antibody sensor functionalization. Continuous detection of IL-6 over 0.2–200 pg/mL in human sweat was demonstrated for a period of 10 hours post-antibody sensor functionalization. Furthermore, combinatorial detection of IL-6 and Cortisol in human sweat was established with minimal cross-talk for 0–48 hours post-antibody sensor functionalization.

## Introduction

Wearable sensors for monitoring biomarkers for chronic health conditions is of significant commercial interest^1^. Sweat based biomarker monitoring with multiple measurements within a 24 hour period are particularly attractive as diagnostic devices^1^. Among the various types of wearable sensors, non-faradaic electrochemical sensors are of particular interest as they enable label-free and non-invasive detection of biomarkers. However, to advance these wearable non-faradaic sensors as diagnostics devices, it is essential to address the challenges of stability and reliability of the materials constituting the sensor during prolonged and continuous exposure to body fluids.

Thus enhancing the stability of affinity based capture probes is critical for the prolonged functionality and performance of wearable diagnostic biosensors^2–5^. The biochemical integrity of these capture probes need to be maintained during the continuous and prolonged exposure to sweat in order to report multiple measurements in a 24 hour period. Several strategies involving surface modification of the sensor and or the capture probes adopted to retain the chemical integrity of the capture probes have been unsuccessful^6^. The key challenge has been the retention of the chemical structure of the capture probes which is essential for achieving stable and repeatable sensing of the target biomarker.

Room temperature ionic liquids (RTILs) have shown to enhance the stability of biomolecules such as proteins and enzymes^6^. This work leverages the method of immobilization of capture probes (antibodies) in a compatible ionic liquid for enhancing stability of antibodies and enable reliable quantification of proteins. We integrate this strategy with the ongoing work in our group with the use of functional nanomaterials towards designing electrochemical biosensors. We aim towards providing a comprehensive solution for wearable diagnostic device applications by ensuring stability and reliability of the bound capture probes at the sensing interface and also in the transduction of the electrochemical signal thereby, enhancing the biosensor performance.

RTILs have been widely investigated for protein extraction, purification, stability and many other applications related to enzymes, amino acids and peptides^2, 7–16^. RTILs are being studied widely due to their desirable properties such as low volatility, wide electrochemical window, and high thermal and chemical stability over conventional solvents^17^. These properties of RTILs can be modulated by the optimal choice of cationic and anionic moiety towards enhancing protein conformational stability. Several studies in this area have concluded that RTILs with low kosmotropic cation and high kosmotropic anion are desired to achieve higher protein stability^18–21^. The disruptions to charge and hydrogen bonding network within RTIL formulations have been evaluated for their antimicrobial and antifungal properties^22, 23^, non-toxicity to cells^24, 25^, and recently for transdermal drug delivery and pathogen neutralization^26^. In this study, we demonstrate for the very first time the use of BMIM[BF_4_] as a stabilizing agent for antibody capture probes immobilized on functional material surfaces suitable for wearable bio sensing.

RTILs have been previously studied for their electrostatic interactions with metal oxide surfaces and the resulting modulations to electrical double layer (EDL) because of their ability for high density charge accumulation^27^. The electrostatic and electrochemical interactions of cations and anions of RTILs with semiconducting ZnO impart large interfacial capacitance that can also be used for signal amplification^27, 28^. We evaluated the stability of the ZnO nanoporous sensor arrays towards validation of RTILs through the detection of interleukin-6 (IL-6) biomarker in human sweat.

IL-6 is an inflammatory pluripotent cytokine comprising of 212 amino acids and is secreted by lymphoid and non-lymphoid cells. IL-6 is an important biomarker and can be potentially used in monitoring immune response in treatment of cancer^29^. Increase in IL-6 levels has also been associated with elevated levels of acute stressors and cortisol secretion during psychological stress after meta-analysis^30, 31^. Moreover, IL-6 increases basal glucose intake and can influence insulin activity^32^. There is a tremendous value in monitoring IL-6 levels. As unlike other biomarkers, levels of IL-6 remain significantly similar in plasma and sweat i.e. 5–15 pg/mL^33^. We demonstrate the use of BMIM[BF_4_] RTIL for enhancing the stability of sensors using capture probe immunoassay functionalized ZnO thin films deposited on nanoporous polyamide membrane. Temporal studies for evaluating the stability of sensor performance for detection of IL-6 from human sweat was carried out using Fourier transform infrared spectroscopy (FTIR), X-ray photoelectron spectroscopy (XPS), Dynamic Light Scattering (DLS), and electrochemical impedance spectroscopy (EIS).

## Results and Discussions

This section is organized as follows: (1) Structural characterization of functionalized ZnO sensor arrays fabricated on nanoporous polyamide substrates; (2) ATR-IR spectroscopy analysis of efficacy and stability of biomolecule binding in RTIL; (3) DLS analysis of protein stability in RTIL using hydrodynamic radius and zeta potential; and (4) Sensor analytical performance and cross-reactivity studies using EIS.

### Structural characterization of linker functionalized ZnO sensor arrays on nanoporous polyamide substrates

**Figure 1:**
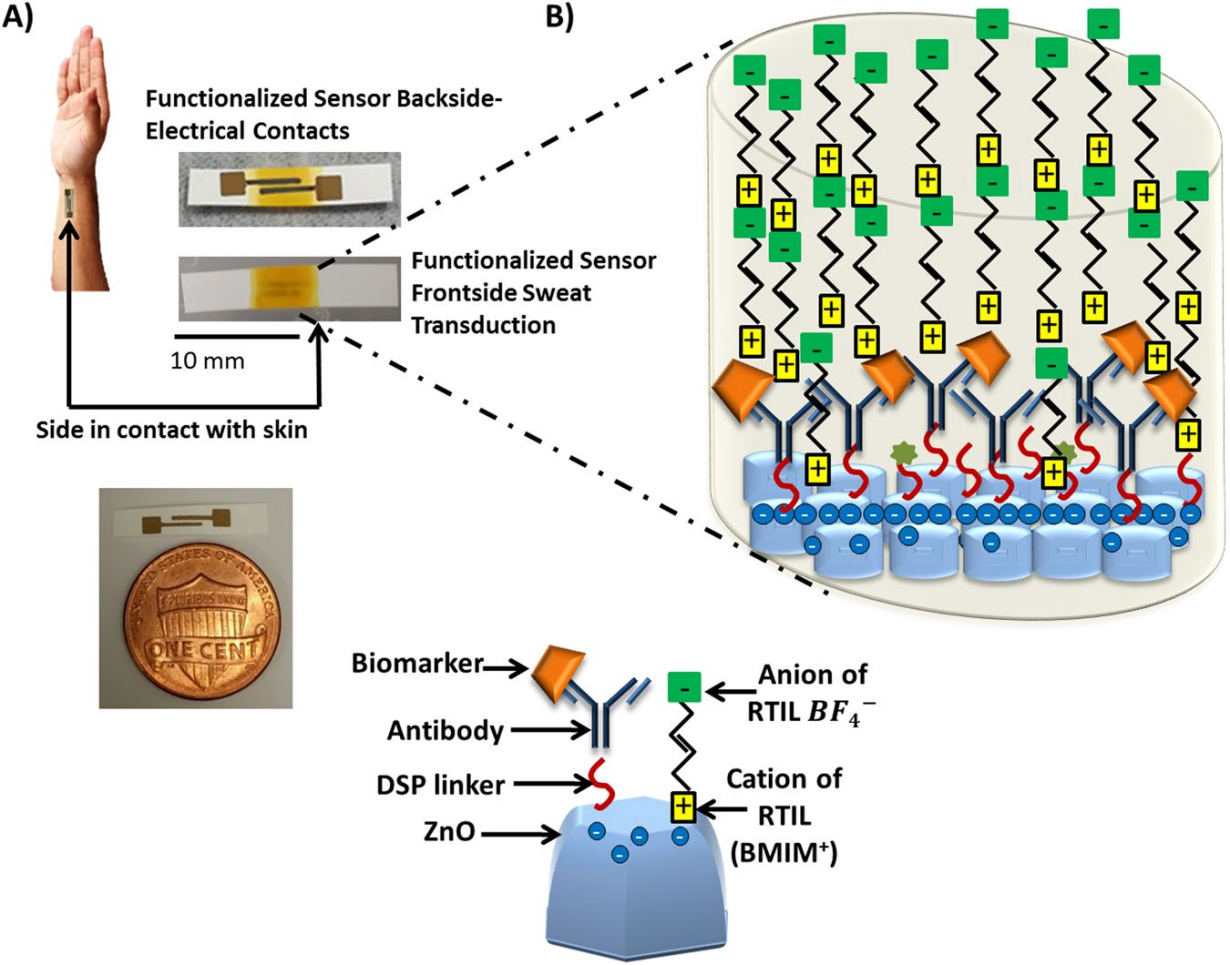
(**A**) Schematic showing our vision of a wearable diagnostic sweat based biosensing and relative size of the developed sensor with RTIL and immunoassay functionalized semiconducting ZnO films on nanoporous polyamide substrates. (**B**) Image showing restricted wicking of fluid in active region of sensor along with a schematic showing capture probe–target biomarker interaction in RTIL and immunoassay with ZnO thin film on a porous membrane within the wicked region of the fluid.

**Figure 2:**
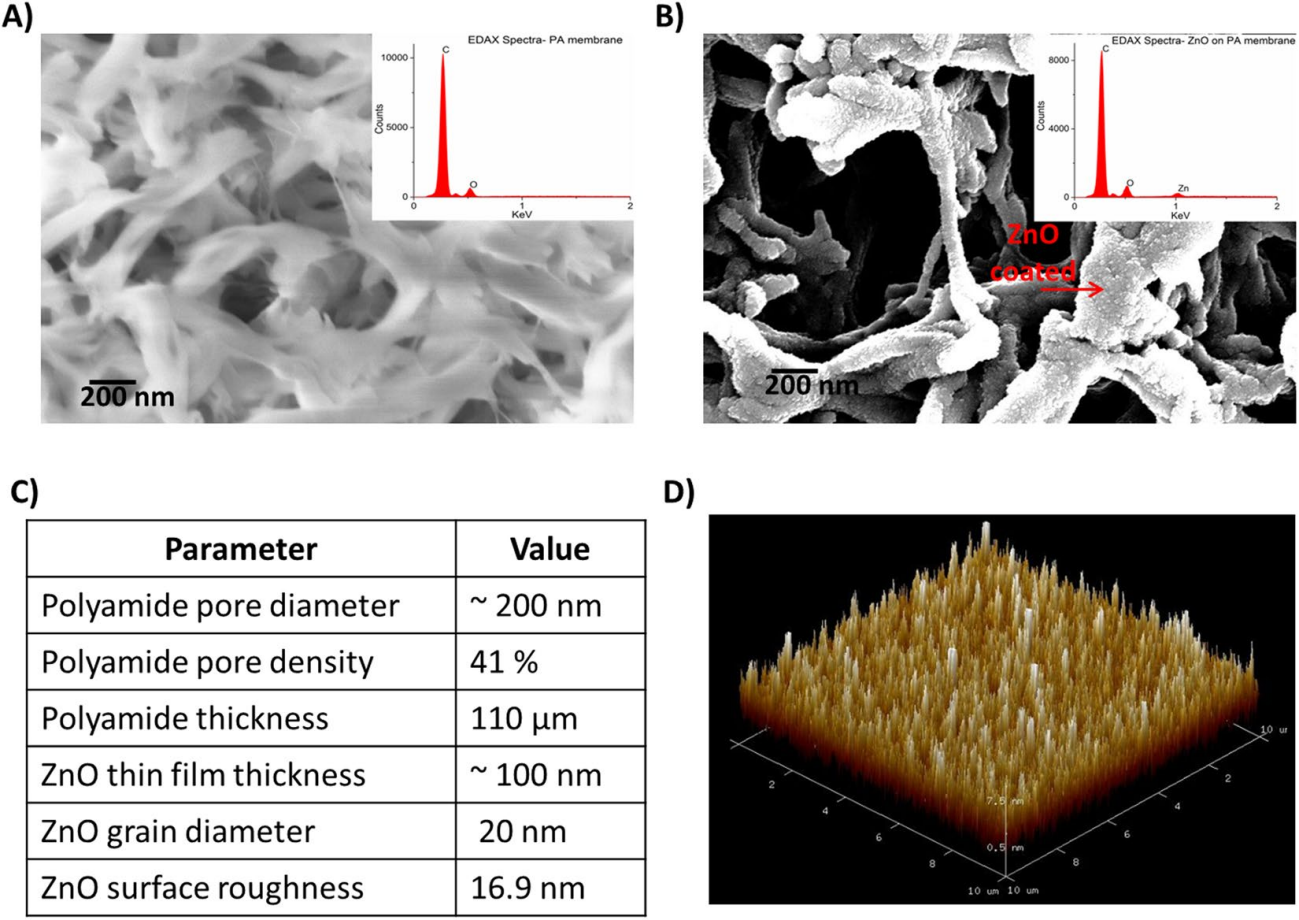
(**A**) SEM of nanoporous flexible membrane with inset showing EDAX spectra of the polyamide membrane surface. (**B**) SEM of ZnO sputtered polyamide membrane with inset showing EDAX spectra of the membrane surface. Zn peaks are only seen on the sputtered polyamide surface. (**C**) Structural parameters of membrane and ZnO thin film D) AFM image of ZnO thin film on silicon wafer.

Perspiration for a normal human being on palm and fingers is estimated to be 5–10 nL/min/gland^34, 35^ and therefore it is essential that the sensor array is capable of operating at these small volumes of absorbed sweat when it comes in contact with the skin. We used nanoporous polyamide membranes as the substrate material for fabricating ZnO sensor arrays and optimized the process for absorption and transport of these small volumes of sweat to the sensor surfaces. The human sweat volumes were maintained between 3–5 µL for all the dose concentration related studies to allow us to correlate the results across methods and samples in this publication. Figure 1A shows the wicking of 3–5 µL of buffer solution on active region of the sensor. In our sensing design and method, the introduction and transduction of sweat is done on the face of the porous polyamide membrane opposite to that of the face on which the sensing material and electrode stack are fabricated as shown in Fig. 1A. Sensing is done through analyte binding to antibody bound sensing electrode surfaces as shown schematically in Fig. 1B. The implications of this sensor design, results in a size based selection of molecules being presented to the sensing electrode surfaces. Thus, an enhanced SNR can be achieved for small analyte biomolecules such as metabolites, protein, enzymes, etc. detection on the sensing electrode surfaces and that would not have been possible without the size based selection. Figure 2A,B show the SEM images with EDAX spectrum in inset for the polyamide membrane and the ZnO sputtered polyamide membrane respectively. The intercalated nanoporous structure of the membrane is evident from these images. A peak corresponding to Zn L-shell is observed at 1 KeV for the ZnO sputtered polyamide membrane only. Thus, indicating a uniform coating of ZnO film on the porous polyamide membrane. Figure 2C lists the structural parameters of the polyamide membrane from the vendor^36^. The sweat travels through the 110 µm membrane thickness and 200 nm nominal pores containing the RTIL to reach the sensing electrode surfaces. Using ATR-IR spectroscopy as described in the next section, we have established there is no hindrance of sweat analyte diffusion in the RTIL wicked membrane. The AFM characterization of the sputtered ZnO thin film on silicon substrate revealed a surface roughness of 16.9 nm and grain diameter of 20 nm. Thus a nanotextured ZnO surface is expected for the thin film deposited on the nanoporous polyamide membrane.

**Figure 3:**
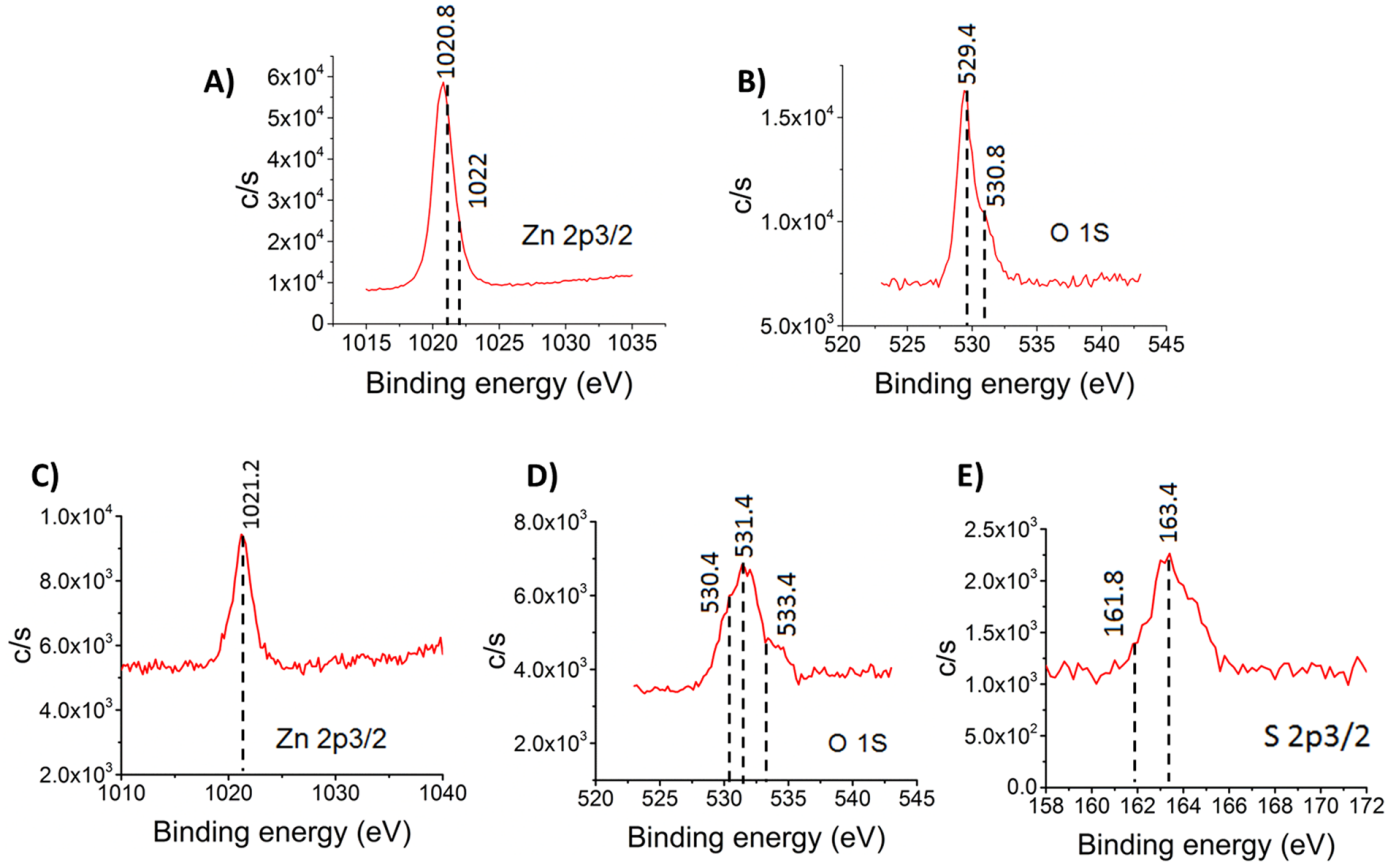
XPS plots of (**A**) Zn 2p_3/2_ peak (**B**) O 1 s peak in bare ZnO and (**C**) Zn 2p_3/2_ peak (**D**) O 1 s peak (**E**) S 2p_3/2_ peak in DSP functionalized ZnO substrate.

XPS analysis was performed to establish the successful binding of thiol based DSP linker on ZnO surfaces. The atomic composition of ZnO thin film was estimated to be 54% Zn, 31% O and 12.6% C. A comparison of the XPS spectra measured from bare ZnO and thiol-functionalized ZnO thin film is shown in Fig. 3. Figure 3A and B show Zn 2p_3/2_ peak at 1020.8 eV, 1022 eV and O 1 s α and β peaks at 529.4 eV, 530.8 eV respectively in bare ZnO thin film. Figure 3C,D and E show Zn 2p_3/2_, O 1 s α, O 1 s β and S 2p_3/2_ peaks of DSP functionalized ZnO surface. Post functionalization with DSP linker, the thiolated Zn 2p_3/2_ peak is shifted to 1021.2 eV indicating displacement at the surface due to Zn–S bonds. The O 1 s peaks are shifted to 530.4 eV and 531.4 eV while a peak at 533.4 eV indicated the presence of adsorbed –O-H species. The S 2p_3/2_ peaks are found to be at 161.8 eV and 163.4 eV, which indicated presence of reduced sulfur as expected for bound thiol moiety to Zn terminations^37–39^. The absence of peak at ~170 eV in spectrum confirmed the absence of oxidized sulfur moiety.

### ATR-IR spectroscopy analysis of efficacy and stability of biomolecule binding in RTIL

While X-ray crystallography and NMR spectroscopy provide the greatest level of detail about a protein’s structure, IR spectroscopy, can also be effectively and readily applied to understanding protein interactions and stability in RTIL-wicked nanoporous polyamide membranes.

### Diffusion of biomolecules in RTIL wicked nanoporous membranes

**Figure 4:**
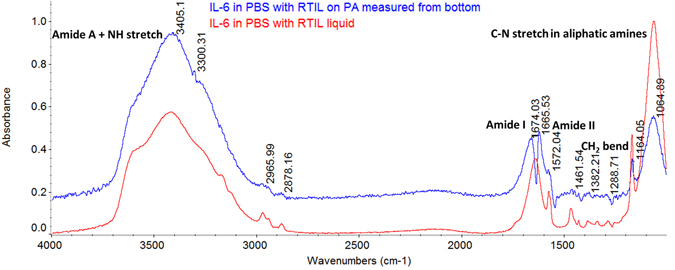
FTIR of polyamide surface on the side directly opposite to the surface used for applying the RTIL with IL-6 solution. The spectra establish no hindrance to the wicking and diffusion of IL-6 in RTIL through the nanoporous membrane thickness.

1-butyl-3-methylimidazolium tetrafluoroborate (BMIM[BF_4_]) RTIL has cation and anion groups that have been used for protein binding, characterization and purification^19, 20^. BMIM^+^ cation and BF_4_^−^ anion both fall at the center of kosmotropic and chaotropic region of Hofmeister series respectively^21^. Thus, they serve as a good starting point for evaluating the effect of RTIL on protein stability^40^. The RTIL BMIM[BF_4_] (94.89 cP) used in this study is more viscous than PBS buffer (1 cP). Figure 4 shows the ATR-IR spectrum of liquid drop (3–5 µL) of IL-6 antigen diluted in RTIL measured on the top and bottom of polyamide membrane immediately after IL-6 in RTIL is dispensed. The expected peak positions typical of protein structures are listed in Table 1. The peaks associated with Amide A due to N-H stretching vibration is observed at 3300 cm^−1^ in both the spectra. Amide I peaks due to stretching vibration of C=O are observed at 1674 cm^−1^ and 1665 cm^−1^ respectively. Amide II peaks derived mainly from in-plane N-H bending are observed at 1572 cm^−1^. C-N stretch of aliphatic amines^40^ is also observed at 1064 cm^−1^. These results conclude the diffusion of protein through the nanoporous polyamide membrane without any hindrance in presence of RTIL.

**Table 1:** Expected IR peak positions pertaining to the secondary structure of proteins.

Description	Expected Peak position
C- H stretch of alkane	3000–2640 cm^−1^
CH_2_ bending	1465 cm^−1^
CH_3_ bending	1375 cm^−1^
CH_2_ (four or more) rocking	720 cm^−1^
C-N stretch in aliphatic amine	1020–1250 cm^−1^
Amide A, N-H stretch	3225–3280 cm^−1^
Amide I	1600–1700 cm^−1^
Amide II	1510–1580 cm^−1^

### Temporal stability of ZnO bound DSP linker–target antibody conjugation in RTIL

**Figure 5:**
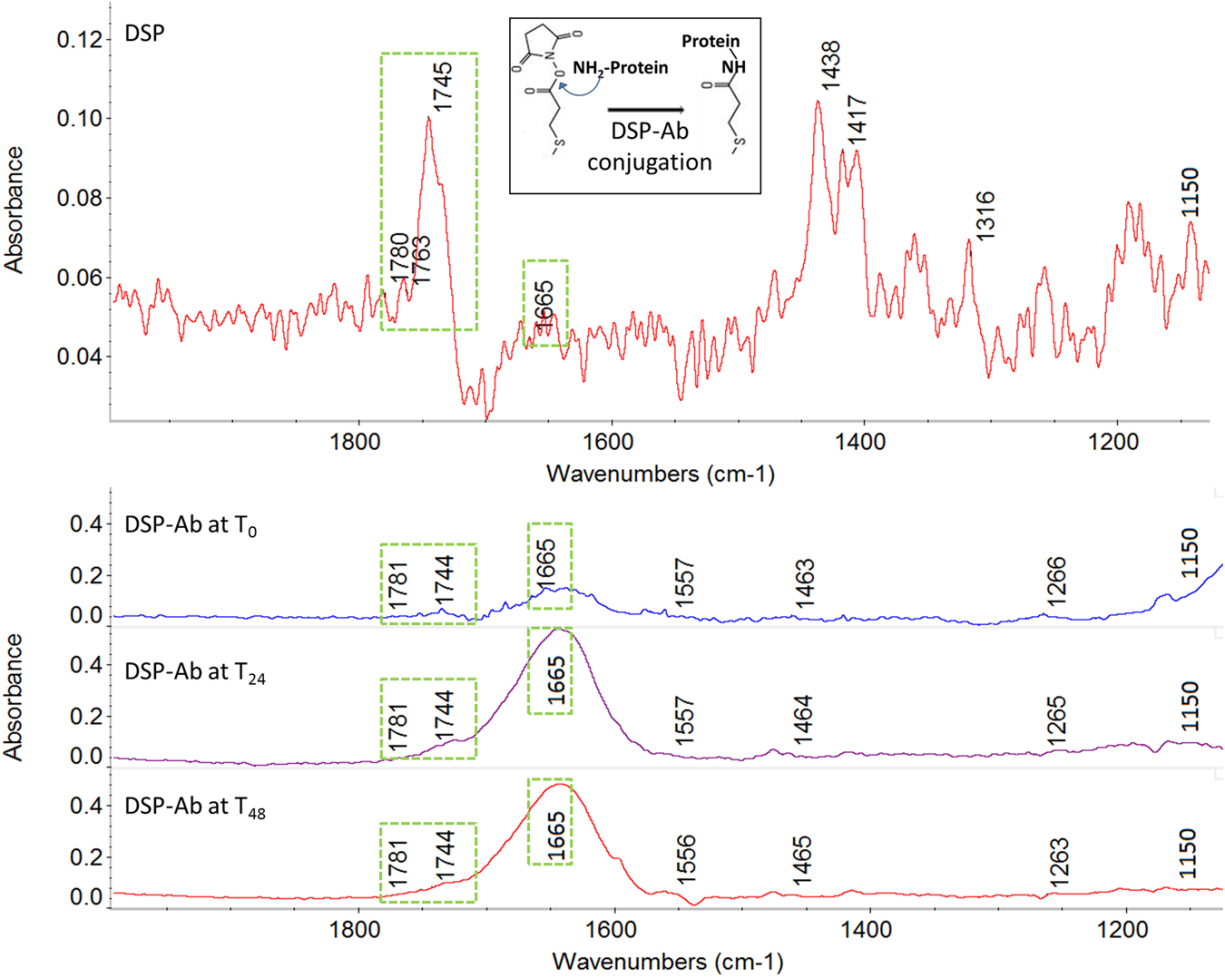
FTIR spectra of DSP linker immobilized on ZnO surface (top), α-IL-6 antibody immobilized on DSP linker after T_0_, T_24_ and T_48_ hours (bottom).

Figure 5 shows the ATR-IR spectra of functionalized ZnO surface with DSP linker and α-IL-6 antibody spiked in RTIL over a period of 48 hours. The first spectrum shows the immobilization of DSP linker onto the ZnO surface. Peaks in 1777–1781 cm^−1^ confirm the presence of symmetric carbonyl stretch of NHS ester in DSP. Peaks at 1762 cm^−1^ and 1744 cm^−1^ are due to asymmetric carbonyl stretch of NHS in DSP, while peak at 1742 cm^−1^ indicates free carboxylic acid. Peaks at 1438 cm^−1^ and 1417 cm^−1^ indicate vibrations of methylene scissors in DSP. Symmetric C-N-C stretch of NHS is observed at 1316 cm^−1^ ^38^. Other spectra show the DSP linker bound protein antibody over a period from 0–48 hours. The binding between DSP linker and antibody is indicated by the breaking of C-O bond of NHS ester and binding of primary amine of the antibody in that position depicting aminolysis as shown in inset of Fig. 5. This process is verified through: 1) decreasing peak height of 1780 cm^−1^ and increasing peak height at 1665 cm^−1^ from DSP only spectra to DSP-antibody spectra at T_0_, T_24_ and T_48_ hours respectively; and 2) breaking of C-O vibrations that is indicated by decreasing peak at 1150 cm^−1^ over time. These changes in peak heights is due to the cleaving of NHS ester bond in the DSP due to the resulting conjugation with the antibody forming a stable Amide bond that is confirmed with the increase in Amide I peaks. The assignments and peak heights are listed in Table 2. These results show a stable and reliable binding of the capture probe antibody to the DSP functionalized ZnO surface in the presence of RTIL within the nanoporous polyamide membranes.

**Table 2:** Band assignments in DSP linker (blue) and in antibody (red) with their corresponding peak position and peak heights (*relative values).

Assignment	Description	Expected Peak Position (cm^−1^)	Parameter	DSP functionalized surface	Antibody IL-6 in RTIL		
					T_0_	T_24_	T_48_
ν_s_(C=O)	Symmetric carbonyl stretch of NHS	1780	Peak position	1777	1779	1779	1779
			Peak height*	0.012	0.002	0.002	0.002
ν_a_(C=O)	Asymmetric carbonyl stretch of NHS	1750–1675	Peak position	1762/1744	1744	1744	1744
			Peak height*	0.013/0.055	0.007	0.041	0.033
ν_s_(C=O)	Free carboxylic acid	1742	Peak position	NA	1744	1744	1744
			Peak height*	NA	0.007	0.041	0.033
ν(C=O)	Amide I	1665	Peak position	NA	1667	1664	1666
			Peak height*	NA	0.083	0.411	0.346

Deconvolution of Amide I (1720 cm^−1^ to 1600 cm^−1^) peaks provides information on α-helix, β-sheet and turn structures of the secondary structure of proteins. This information can be quantified to understand the stability of secondary structure of protein. The de-convolved spectra for time intervals from T_0_ to T_96_ hour storage of antibody in BMIM[BF_4_] and PBS are displayed in Supplementary in Fig. S1. It was observed that there were significant structural conformational changes occurring for antibody dissolved in PBS in comparison to that of antibody dissolved in RTIL. The degradation in α-helix and β-sheet structures is prominent starting only at T_96_ hours for antibody dissolved in RTIL, while the degradation had occurred well before T_48_ hours for antibody dissolved in PBS. In summary, the ATR–IR spectroscopy analysis of the immunoassay steps clearly establishes the unhindered conjugation of DSP-antibody in the presence of RTIL and that the RTIL buffer environment provides stability of the bound protein across time and environment for upto 96 hours in comparison to less than 48 hours for the same bound protein in PBS buffer.

### DLS analysis of protein stability in RTIL using hydrodynamic radius and zeta potential

ATR-IR spectroscopy provides avenues to analyze the stability of unbound and bound proteins in buffers in reference to its primary and secondary structures. The charge states and electrophoretic behavior of both bound and unbound proteins suspended in solution is also affected by the properties of human sweat such as varying pH, temperature, ionic composition, and water content. The hydrodynamic size (R_h_) measured using dynamic light scattering (DLS) and zeta potential trends therefore can be used to understand the charge state and the electrophoretic characteristics of the unbound/bound proteins in various solutions. The principle of operation of the DLS is based on Brownian motion of particles, where the mobility and diffusivity of the target protein in solution is related to their size and interactions with the other particles present in the solution. Depending on the quantity and polarity of the charge on the particles, the proteins will either tend to flocculate or resist flocculation depending on the composition and nature of the solvent resulting in an EDL formed at the protein surface. Therefore, under an applied electric field, the surface charge acquired by the protein in suspended solution can be characterized through zeta potential measurements. Furthermore, the surface charge characteristics and mobility of the unbound and bound states of the protein under applied field result in the modulation of the EDL. This when measured using electrochemical methods such as EIS and CV can be used as electrical sensing modalities for affinity based detection of biomarkers.

**Figure 6:**
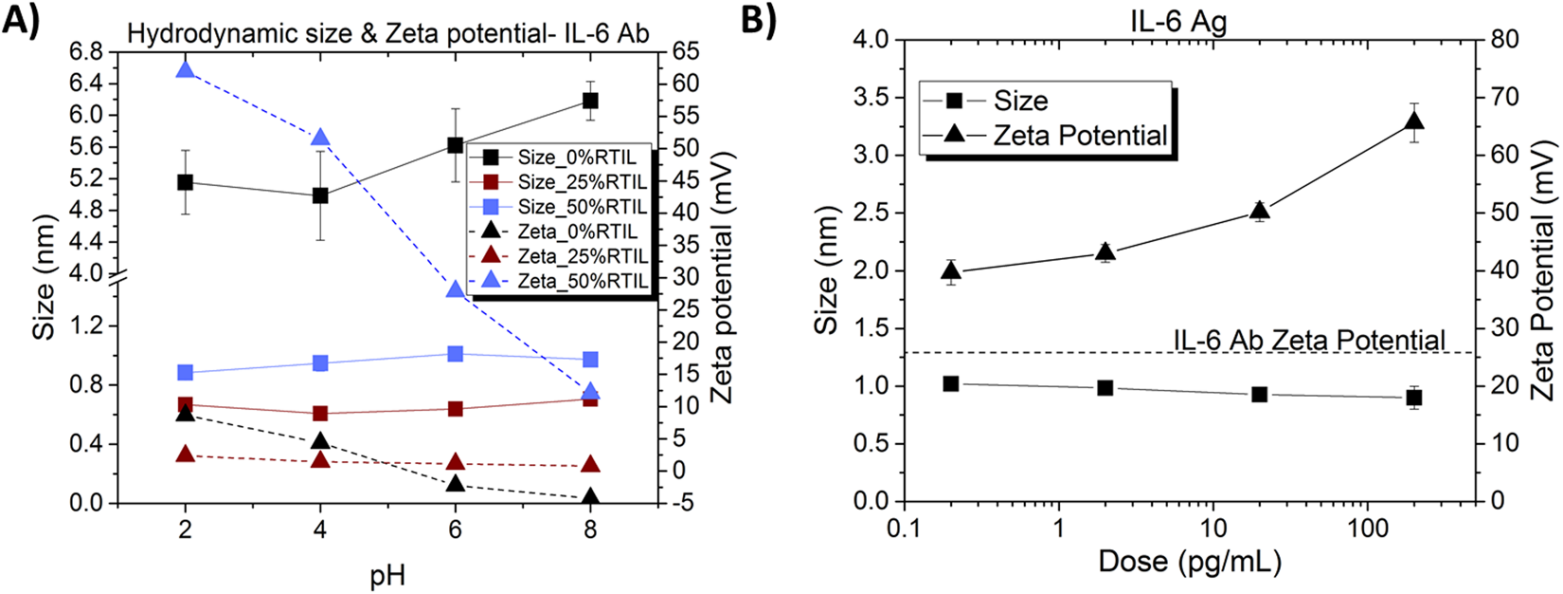
DLS and zeta potential measurements. (**A**) α-IL-6 antibody spiked in synthetic sweat (SS) of varying pH and RTIL ratios; (**B**) IL-6 antibody-antigen interactions in 50% RTIL in SS solutions. The dotted line in (**B**) represent the zero dose antigen zeta potential value for the respective proteins.

In this DLS study, we evaluated the stability of protein capture probe molecules α-IL-6 antibody (Fig. 6) and α-cortisol antibody (Supplementary Fig. S3). These biomolecules were individually suspended in a mixture with variable volumetric ratio of RTIL and synthetic sweat (SS) of pH 2, 4, 6, and 8. Figure 6A represents the R_h_ and zeta potential of IL-6 antibody in 0%, 25%, 50% RTIL in SS of pH 2 to pH 8. It can be observed that the R_h_ of α-IL-6 dissolved in 0% RTIL increases from 5 nm to 6.4 nm as the pH of SS varied from pH 2 to 8. This indicates that the protein size is a function of pH in pure aqueous buffer without any RTIL. However, for 25% RTIL and 50% RTIL mixture solutions, the R_h_ of α-IL-6 is constant at 0.7 nm and 0.9 nm respectively with varying pH of SS. Similar results were obtained when R_h_ of α-cortisol as demonstrated in Supplementary Fig. S3A. Thus, for greater than 25% RTIL volumetric ratio in SS solutions, the protein’s structural conformational stability is maintained as represented by the constant hydrodynamic size for varying pH ranges. The size R_h_ of the suspended protein antibodies increases slightly in presence of higher RTIL concentration (≥50%). This can be caused due to formation of compact layers of cation and anionic moieties of RTIL surrounding the protein protecting it from hydrolysis. The protein aggregation normally observed close to isoelectric point was inhibited in these higher RTIL concentration solutions imparting conformational stability to the proteins.

The zeta potential measurement of α-IL-6 in 0% RTIL solution as demonstrated in Fig. 6A varies from 7.5 mV to -5 mV across pH range 2 to 8, with the crossover occurring at pH 5. The zeta potential stays constant across pH at 1 mV for 25% RTIL solution, while at 50% RTIL the zeta potential varies from 62.5 mV to 10 mV across pH range from 2 to 8. Similar results were obtained for α-cortisol as demonstrated in Supplementary in Fig. S3A. The zeta potential of proteins in 25% RTIL does not vary significantly across the pH range. This might be due to formation of aggregates or sedimentation due to absence of strong polarity between the particles and is likely to cause destabilization of proteins over time due to flocculation or aggregation. At 50% RTIL, the zeta potentials are higher in magnitude and has a 4–5X wider dynamic range (i.e. max-min across pH range from 2 to 8) compared to 0% RTIL. The protein does not display neutral state (i.e. point of zero charge) for this pH range. Since particles with similar surface charge states and higher magnitude will repel each other strongly, they will resist flocculation or aggregation imparting enhanced conformational stability. This explains the stability of protein antibodies suspended at higher RTIL concentrations. The higher and stable zeta potentials of the suspended protein antibodies in ≥50% RTIL solutions is also indicative of a strong and energetically active EDL formed around the protein surface. It results in the proteins responding to small changes in external applied fields. This phenomenon can therefore be easily translated to electrochemical sensing methods that rely on changes to EDL for detection and quantification of biomolecular events.

We evaluated the antibody-antigen interactions and binding events to ensure the functionality of the sensing is not adversely hindered in the presence of RTIL due to the higher magnitude of zeta potentials. Figure 6B represents the IL-6 antibody-antigen interactions in 50% RTIL in SS solution for varying doses of antigen. The R_h_ of molecules remains constant at 1 ± 0.2 nm and zeta potentials increases from 40 mV to 70 mV with increasing antigen dose concentrations. As zeta potentials are a representation of electrophoretic mobility^41^, the increasing antigen dose concentrations would result in a higher surface charge around the antibody-antigen conjugate to repel against similar charge. Thus increasing the mobility of the conjugate and subsequently increasing the zeta potential. Similar results for cortisol antibody-antigen interactions are demonstrated in Supplementary Fig. S3B. This increase in zeta potential at constant R_h_ with increasing antigen concentration also indicates the enhanced magnitude of charge within the EDL resulting from the antibody-antigen conjugation, while preserving the conformational state of the protein thus ensuring stability of protein for over 96 hours in synthetic sweat solutions with ≥50% RTIL concentrations.

### Sensor analytical performance and cross-reactivity studies using EIS

#### EIS calibration of antigen IL-6 detection in human sweat

**Figure 7:**
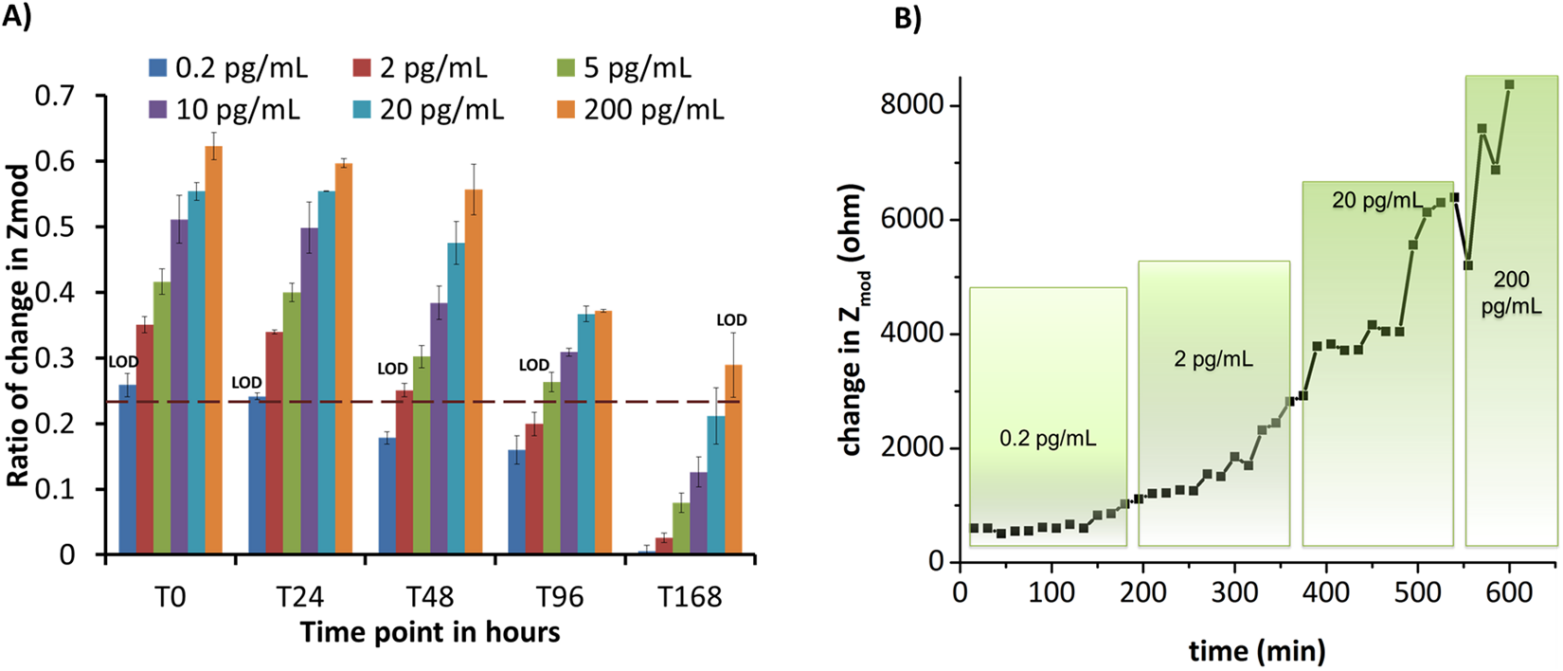
(**A**) Percent change in Zmod ratio of antigen IL-6 concentration varying from 0.2 pg/mL to 200 pg/mL tested for varying storage time of antibody IL-6 for time T_0_, T_24_, T_48_, T_96_ and T_168_ at 4 °C. The dashed line represents specific signal threshold (SST). (**B**) Continuous time based detection of IL-6 in human sweat through impedance change.

Non-Faradaic EIS measurements quantify the binding interactions of immunoassay and antigen based on mainly the capacitive changes that occur at the electrical double layer (EDL) due to change in dielectric constant. The EIS data analyzed using Nyquist and Bode plots is described in the Materials and Methods section. The binding of antibody IL-6 with antigen IL-6 was measured using EIS. Figure 7A represents the ratio of change in Z_mod_ captured at 10 Hz for a particular condition. The ratio was calculated as per equation 1 below for the concentration of antigen IL-6 in human sweat from 0.2 pg/mL to 200 pg/mL. Each of the datasets shown on x-axis represents the time of storage of antibody functionalized sensor surface at 4 °C i.e. T_0_, T_24_, T_48_, T_96_ and T_168_ hours post antibody IL-6 immobilization and prior to addition of antigen IL-6 doses in human sweat. The SST calculated as described in Materials and Methods section was found to be at 0.22 which is indicated by the dashed brown line in Fig. 7A. Also, shown in Fig. 7A is the LOD for each calibrated datasets.

1 $$ratio\,of\,change\,in\,{Z}_{mod}=\frac{({Z}_{mod}\,of\,concentration-{Z}_{mod}of\,baseline)}{{Z}_{mod}of\,baseline}$$

At T_0_, the ratio varies from 26% to 62% for increasing concentration of antigen IL-6 in human sweat from 0.2 pg/mL to 200 pg/mL. At T_24_ and T_48_, the ratio varies from 24% to 60% and from 18% to 56% respectively for varying concentration of antigen IL-6 in human sweat from 0.2 pg/mL to 200 pg/mL. At T_96_ and T_168_, the dynamic range is 16% to 37% and 5% to 29% respectively. This sudden drop in the ratio of change in Z_mod_ for T_96_ and T_168_ is attributed to reduced binding between antigen to antibody likely due to the deformations to the conformational structure of the protein antibody after 96 hours storage time. Since the antibody-antigen interaction is weakened, the changes in dielectric constant at EDL for lower doses are not well quantified above the noise level of the sensor system. The EIS results correlate well with the results of deconvoluted ATR-IR spectra analysis of antibody presented in Supplementary that also indicate α-helix and β-sheet coverage under Amide I peak are reduced drastically after T_96_ hours storage time. The binding site between antigen IL-6 and antibody IL-6 is located at the C-terminal side of gathering of α-helical structures^42^. The decline in α-helical structure’s contribution in Amide I peak post T_96_ hours correlates to the reduction in ratio of change in Z_mod_ at T_96_ and T_168_ hours. Thus, the LOD changes from 0.2 pg/mL for T_0_ and T_24_ hours, to 2 pg/mL for T_48_ hours, to 5 pg/mL for T_96_ hours, and to 200 pg/mL for T_168_ hours respectively.

When antibody IL-6 dissolved in BMIM[BF_4_] is incubated on DSP linker functionalized ZnO surface, an EDL is formed as represented in Fig. 1B. Kosmotropic anion BF_4_^−^ and less kosmotropic cation BMIM^+^, when present in high concentration (~75% by volume ratio in this EIS measurement) tend to form compact structures around the protein, protecting it from hydration and preventing hydrogen bond formation in aqueous solutions^21^. Hence, after antibody binding to DSP linker, the anion BF_4_^−^ will surround the antibody forming a core around antibody. This anion will then be surrounded by cation and so on, forming a multilayer EDL structure until electrostatic and thermodynamic equilibrium is achieved. This multilayer EDL structure thus formed provides stability to the protein antibody while also increasing the capacitance of the system due to the electrochemical nature of RTILs. Therefore, modulations to EDL due to target binding events can be reliably characterized using capacitance changes in EIS measurements enabling detection and quantification of antigen IL-6 in human sweat using the sensor.

We also evaluated the robustness of the sensor for real-time monitoring by performing continuous detection of IL-6 in human sweat. Incrementally varying dose concentration of IL-6 between 0.2 pg/mL to 200 pg/mL in human sweat were dispensed onto the sensor incubated with antibody IL-6 (T_0_ condition). The doses were introduced sequentially in 30 minute intervals for a total experiment duration of 10 hours (limited by the laboratory setup maintained at ambient room temperature). Change in total impedance Z_mod_ was calculated at fixed frequency with respect to baseline for each of the concentration doses as shown in Fig. 7B. The results indicate constant change in impedance of 660 Ω with respect to baseline for all 0.2 pg/mL concentration doses, 1100–2800 Ω for all 2 pg/mL concentration doses, 4000–5000 Ω for all 20 pg/mL concentrations and above 6000 Ω for all 200 pg/mL concentrations. Constant change in impedance for a specific IL-6 concentration dose and incremental changes in impedance proportional to increased IL-6 concentration doses indicate stable and reliable real-time, continuous detection of the RTIL sensor for IL-6 detection in human sweat. Although, this experiment was limited to a 10-hour duration, the change in impedance with doses indicate a longer continuous detection sensor performance considering that the established clinical range for IL-6 in human sweat is 7–16 pg/mL.

### Specificity of antigen IL-6 detection in human sweat

**Figure 8:**
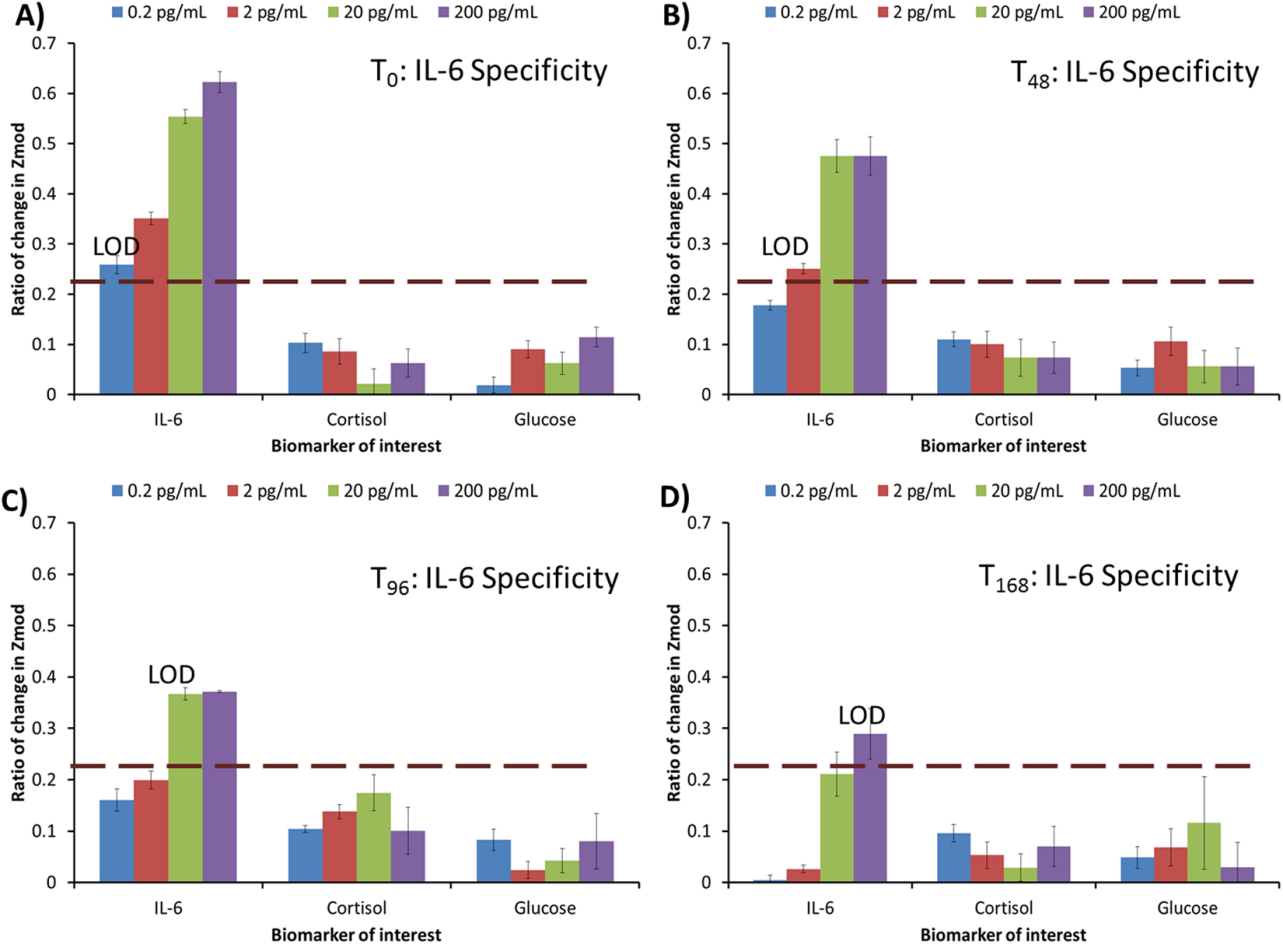
Specificity of antibody IL-6 interactions towards antigen IL-6 and non-specific interactions with cortisol and glucose biomolecules for time intervals of antibody functionalized ZnO sensors stored at T_0_ to T_168_.

Specificity requires the sensor to respond only to the target analyte and not to other similar molecules. Label-free biosensors depend on antibody or capture probe selectivity to distinguish between specific and non-specific interactions. Thus in order to evaluate the specificity of the immunoassay developed for IL-6 detection, experiments were performed in the presence of cortisol and glucose molecules as described in Materials and Methods section. Figure 8 displays the calibration for cortisol, glucose, and IL-6 molecules each varying in range of 0.2 pg/mL to 200 pg/mL tested on antibody IL-6 functionalized ZnO sensor arrays for T_0_, T_48_, T_96_, and T_168_ hours respectively. Significantly higher specific signal above the SST ratio of 0.22 was found for IL-6 detection until T_96_ hours storage time. At all times, the ratio of impedance change for non-specific interactions of cortisol and glucose with IL-6 antibody is found to be well below the SST ratio.

### Combinatorial cortisol detection and specificity in human sweat using EIS

We performed experiments using cortisol antibody for cortisol detection, in order to understand the versatility of developed sensor using RTIL bioimmunoassay functionalized and demonstrate the combinatorial detection performance and protein stability of the developed sensor. The results of this experimentation are described in Supplementary and results plotted in Fig. S4. Linear calibration response for cortisol detection until T_48_ hours was observed.

### Summary

This work is the first report to date, based on our assessments of other similar works in the published domain, of the combinatorial detection of IL-6 and Cortisol in Human Sweat. Also the 1st report to date on the enhanced stability and effect over time on biomolecule sensing performance through the use of ionic liquids. Currently, there is no reported published work on the continuous i.e. over a 12 hour periods for the detection of disease biomarkers in human sweat (Supplementary Table S1). The primary reason is due to the difficulty in ensuring the biomolecules (i.e. metabolites, proteins, enzymes, etc.) stability in the complex buffer media such as sweat with widely varying pH levels.

The stability of antibody IL-6 in BMIM[BF_4_] upto 96 hours and its ability to reliably detect specific antigen in human sweat was established using electrochemical impedance spectroscopic, ATR-IR spectroscopic, and DLS techniques. The calibration for antibody based IL-6 detection in human sweat was linear in the range of 2–200 pg/mL with the LOD found to be 0.2 pg/mL for 0–24 hours, 2 pg/mL for 24–48 hours, and 5 pg/mL for 48–96 hours respectively and in all cases well below the physiologically relevant range of IL-6 in human sweat of 7–16 pg/mL. Thus, it can be concluded that the sensor stability is sustained until 96 hours in the presence of RTIL after immobilization of the antibody and can be utilized for detection of IL-6 from human sweat. Furthermore, real-time sensor performance was demonstrated for a period of 10 hours, in a laboratory experimental setup for continuous detection of IL-6 in human sweat. Additionally, the ability of the sensor for specific and combinatorial detection of cortisol in human sweat with a LOD of 10 ng/mL was demonstrated. Linear detection range matching the physiological range of 10–200 ng/mL was achieved even after 48 hours of immobilization of the antibody onto the sensor surface. Therefore, RTIL generally enhances the accuracy in signal for multiple specific marker detection and quantification.

Hence, with this work which provides a novel approach and a platform for addressing these issues, we envision will add to the growing research and development effort to develop stable and reliable diagnostic biosensors that can be integrated onto sweat based wearable device platforms.

## Methods

### Sensor Fabrication

Figure 1 shows the sensor fabricated on a flexible nanoporous polyamide substrate with the electrodes and active region with the fluids wicked onto the immunoassay functionalized ZnO. Figure 1 also provides a schematic showing our vision of a wearable diagnostic sweat based sensing device application using these fabricated sensors. The detailed procedure followed for fabrication of these sensors is described in the prior publication^43^.

## Reagents and Materials

Polyamide substrates with nominal pore size of 200 nm were obtained from GE Healthcare Life Sciences (Piscataway, NJ, USA). The linker molecule Dithiobis [Succinimidyl Propionate] (DSP) and its solvent Dimethyl Sulfoxide (DMSO) were ordered from Thermo Fisher Scientific Inc. (Waltham, MA, USA). The monoclonal α-IL-6 antibody was obtained from Abcam (Cambridge, MA, USA). Full length recombinant IL-6 protein was obtained from Thermo Fisher Scientific Inc. (Waltham, MA, USA). The monoclonal α-cortisol antibody and cortisol hormone (Hydrocortisone) was ordered from Abcam (Cambridge, MA, USA). D-(+)-glucose was obtained from Sigma-Aldrich (St. Louis, MO, USA). Synthetic sweat composition prepared in the laboratory contains uric acid, lactic acid, ammonia, Na, K and Cl ions. The composition for synthetic sweat was adopted from Mathew *et al*.^44^. Concentration of ammonia was varied in the composition to obtain different pH. Synthetic sweat solutions were prepared with pH 2, 4, 6 and 8. Human sweat purchased from Lee biosolutions Inc. (St. Louis, MO, USA) was collected from single human donor. No preservatives were added to this product and it was stored unfiltered at below -20°C. BMIM[BF_4_] was purchased from Sigma-Aldrich (St. Louis, MO, USA). All antigen dilutions were made in human sweat. Antibody was reconstituted in DI water (conductivity 18.5 MΩ.cm) and further diluted in RTIL in required concentrations.

### Experimental setup for ATR-IR spectroscopy

Infra–Red (IR) spectra were obtained using a Nicolet iS-50 Fourier transform infrared spectrometer equipped with deuterated triglycine sulfate (DTGS) detector and KBr beam splitter. Attenuated Total Reflection (ATR) IR spectroscopy was performed using germanium crystal which has a mid-IR range covering 4000 cm^−1^ to 600 cm^−1^ wavelengths. Germanium crystal offers high refractive index of 4.0 and incident angle of 42° and useful for solutions of pH ranging from 1–14. Spectra were collected in nitrogen atmosphere by performing 1024 scans at a resolution of 4 cm^−1^. Samples for FTIR analysis consisted of ZnO thin films on modified glass substrates using sputter deposition at parameters matched with that used for the sensor fabrication. α-IL-6 diluted using 100% PBS and 75% BMIM[BF_4_] with 25% PBS solutions were incubated on the DSP functionalized ZnO surfaces. The substrates were washed with PBS solution and dried with nitrogen for further experimentation. Time based measurements were carried out at T_0_, T_24_, T_48_, and T_96_ where subscript denotes number of hours of storage of samples at 4 °C.

FTIR spectra was also collected on bottom of polyamide membrane dispensed with 3 µL of IL-6 antigen in RTIL and IL-6 antigen in PBS on separate substrates from top of the membrane to confirm the diffusion of IL-6 protein from top of the membrane to bottom. This study was done to validate the wicking ability of sensor substrate attached to human skin with sweat. We also validated transfer and mobility of biomolecules from bottom of sensor substrate to functionalized ZnO region on top of membrane for detection and quantification.

### Experimental setup for XPS

The XPS measurements were done using a PHI 5000 Versa Probe II with a monochromatic Al Kα radiation (hν = 1486.6 eV). All evaluations were taken at a 45° takeoff angle with respect to the sample surface. Spectra were obtained with a 0.2 eV step size and 23.50 eV pass energy. The base pressure in the analysis was 1.6 × 10^−8^ Torr. Samples were prepared as mentioned in section 2.3 until DSP stage on silicon wafer. All binding energies were corrected for the charge shift using the C 2 s peak of graphitic carbon (BE = 284.8 eV) as a reference.

### Hydrodynamic radius and zeta potential measurements

Dynamic Light Scattering (DLS) technique was performed to determine the hydrodynamic radius (R_h_) and electrophoretic light scattering was carried out to measure zeta potential (ζ) of biomolecules, IL-6 and Cortisol antibody. IL-6 and Cortisol antibody was spiked separately in various dilutions of 0%, 25%, 50% BMIM[BF_4_] (by volume) in synthetic sweat of pH 2, 4, 6 and 8. These experiments were carried out using Malvern Zetasizer NanoZS (Malvern Instruments, UK). Distribution fit by volume was implemented to calculate the size and Smoluschowski approximation was used to compute zeta potential from electrophoretic mobility^41^. The hydrodynamic size and Zeta potential was also calculated for IL-6 antibody-antigen and Cortisol antibody-antigen specific interaction in 50% BMIM[BF_4_] diluted in synthetic sweat. Temporal studies at T_0_, T_24_, T_48_ and T_96_ hours was performed to understand the stability of α-IL-6 antibody in 50% BMIM[BF_4_]. The synthetic sweat was maintained at pH 6 to match with the human sweat pH ~6.4 for antibody-antigen interactions during temporal studies. Viscosity measurements were performed using μVisc viscometer (RheoSense, San Ramon, USA).

### Immunoassay protocol for EIS on nanoporous polyamide ZnO sensor arrays

Thiol-linker functionalization was carried out on ZnO surface after dispensing 3–5 μL volume of 10 mM DSP diluted in DMSO and incubated at room temperature. This was followed by 30 min incubation of 100 µg/mL, α-IL-6 antibody diluted in BMIM[BF_4_] at room temperature. In case of temporal studies, after antibody immobilization at room temperature, sensors were stored in 4 °C for 24, 48, 96, and 168 hours. After successful functionalization of antibody diluted in BMIM[BF_4_], 3–5 µL volume of human sweat solution was dispensed onto the sensor strip and EIS measurement was done. This step was considered as zero dose or baseline measurement. Post baseline measurement, IL-6 dilutions made in human sweat in the range of 0.2–200 pg/mL were then serially dispensed in 3–5 µL volume onto the sensor strip with increasing doses from 0.2 pg/mL to 200 pg/mL and EIS measurements were performed. For validating specificity of the sensor, cortisol molecule of concentration varying from 0.2 pg/mL to 200 pg/mL spiked in human sweat and glucose diluted in the range of 0.2 pg/mL to 200 pg/mL in human sweat were also tested separately on the α-IL-6 immobilized sensor surfaces.

EIS measurements were taken by recording current flow using a potentiostat (Gamry Instruments, Warminster, PA, USA) after applying a small AC voltage with a frequency sweep of 1 Hz to 1 MHz. Sensor calibration response was calculated using n = 4 samples. The response to the varying IL-6 concentration was captured in terms of ratio of change in total impedance (Z_mod_) between the baseline step impedance and impedance obtained for that particular concentration. The Z_mod_ was captured at 10 Hz, the highest signal over noise ratio. Specific Signal Threshold (SST) was estimated by measuring replicates of a blank buffer sample and calculating the mean result and standard deviation^45^. A signal over noise ratio of 3 was used as a robust indicator of sensor performance and SST impedance as three times the noise signal was calculated. The noise level was defined as the multiple of standard deviation in average baseline (zero dose) measurement^45^. Limit of detection (LOD) was identified as the lowest IL-6 concentration likely to be reliably distinguished from the SST and at which detection is feasible^45^.

### Statistical analyses

All the data was analyzed using OriginPro. Data is presented as mean ± std.error of mean. The error bars are drawn using the standard error of mean calculated from the number of replicates mentioned used for experimentation.

### Data availability

The datasets generated during and/or analysed during the urrent study are available from the corresponding author on request.

## Electronic supplementary material

Supplementary Information
