# Supplementary material for: A new paradigm in sweat based wearable diagnostics biosensors using Room Temperature Ionic Liquids (RTILs)
Source: Sci Rep. 2017 May 16;7:1950. doi: 10.1038/s41598-017-02133-0 (PMC5434046; doi:10.1038/s41598-017-02133-0)
Supplement: Supplementary file 1 — Supplementary Information [file 41598_2017_2133_MOESM1_ESM.pdf]

A new paradigm in sweat based wearable diagnostics biosensors using Room Temperature Ionic Liquids (RTILs)

Rujuta D. Munje[a], Sriram Muthukumar[b], Badrinath Jagannath[a], Shalini Prasad[a*]

a. Department of Bioengineering, University of Texas at Dallas, Richardson, Texas, 75080, USA.

b. EnLiSense LLC, 1813 Audubon Pond Way, Allen, Texas, 75013, USA.

* Corresponding author: shalini.prasad@utdallas.edu

Deconvolution of ATR-IR spectra for evaluation of stability of antibody in RTIL

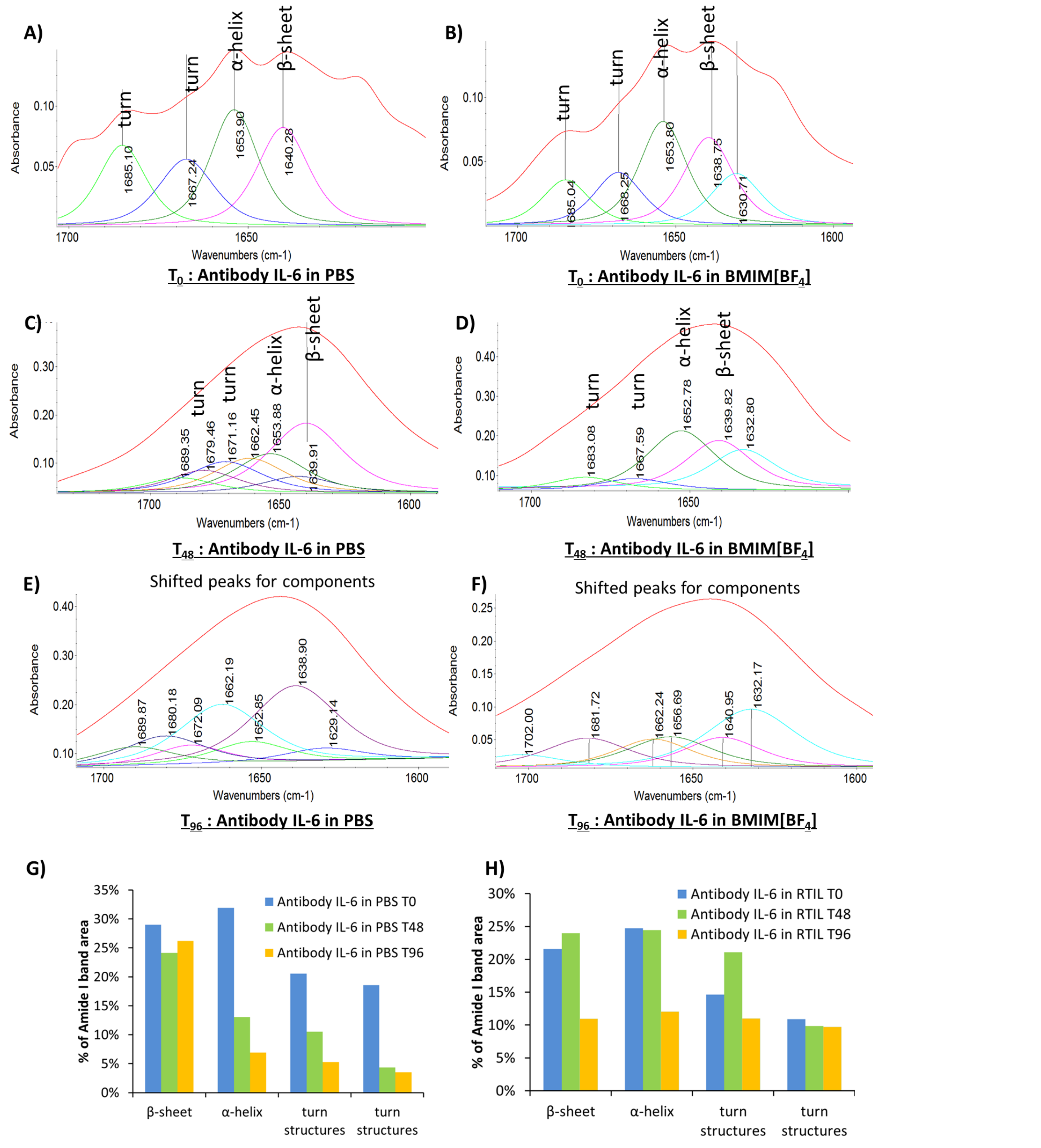

Figure S1. Deconvoluted and quantification of ATR-IR spectra of: A) α-IL-6 antibody in PBS after T0 hours; B) α-IL-6 antibody in RTIL after T0 hours; C) α-IL-6 antibody in PBS after T48 hours; D) α-IL-6 antibody in RTIL after T48 hours; E)

α-IL-6 antibody in PBS after T96 hours; F) α-IL-6 antibody in RTIL after T96 hours; G) quantified percent area of structures of Amide I for α-IL-6 antibody in RTIL; H) quantified percent area of structures of Amide I for α-IL-6 antibody in PBS.

ATR-IR spectroscopy can also provide confirmation of protein secondary structure through characterization of the Amide I band analysis (change in backbone conformation and the hydrogen bonding pattern). The types of protein secondary structures include the α-helices and β-sheets, which allow the amides to hydrogen, bond very efficiently with one another. Both are periodic structures. In an α-helix the polypeptide backbone is coiled in a right-handed helix where the hydrogen bonding occurs between successive turns of the helix. In β-sheets, the strands of polypeptide are stretched out and lay either parallel or antiparallel to one another. The hydrogen bonds form between the strands. The other elements of a protein secondary structure include β-turns and unordered structures. β-turns are sharp turns that connect the adjacent strands in an antiparallel β-sheet. Unordered structures are generally a catch-all for regions that do not fall into one of the other categories [1]. These are often loops which form near the surface of proteins and join the other elements of secondary structure. Thus characterizing the secondary structure of the protein provides us with assessing the state of protein stability.

Deconvolution of Amide I (1720 cm^{-1} to 1600 cm^{-1}) peaks provides information on α-helix, β-sheet and turn structures of the secondary structure of proteins. This information can be quantified to understand the stability of secondary structure of protein. The deconvolved spectra for time intervals from T_0 to T_{96} hour storage for antibody in BMIM[BF_4] and PBS are displayed in Figure S1. It can be observed from the spectra that wavelength corresponding to α-helix (1653 cm^{-1}), β-sheet (1636 cm^{-1}) and turn structures (1669 $cm^{-1}$and 1684 cm^{-1}) are observed for T_0, and T_{48} hours for both PBS and BMIM[BF_4] diluted antibody. However, these peaks were shifted at T_{96} hours for both PBS and BMIM[BF_4] diluted antibody. In order to quantify the change in stability of antibody over time T_0 to T_{96} hours peak area quantification was performed. The peak area calculated as a percentage with respect to

area of Amide I peak for T_0 to T_{96} hours are listed in Figure S1G and Figure S1H for α-helix, β-sheet and turn structures for IL-6 antibody in BMIM[BF_4] and PBS respectively.

The percentage of area pertaining to α-helix (1653 cm^{-1}) for antibody in RTIL decreases from 25% to 12% from T_0 to T_{96} hours. Similarly, the decrease for β-sheet (1636 cm^{-1}) is from 24% to 11% for the same time periods. The percent area of turn structures is similar during the time periods as measured for wavelength 1669 cm^{-1} and 1684 cm^{-1} respectively, except for one outlier at T_{48} hours. However, the area for α-helix (1653 cm^{-1}) decreases from 32% to 5% and for β-sheet (1636 cm^{-1}) from 29% to 26% for antibody in PBS for the same time periods of T_0 to T_{96} hours. The area for turn structures also consistently decreases from 21% to 11% and 19% to 4% for wavelength 1669 $cm^{-1}$and 1684 cm^{-1} respectively. This quantification establishes that there is a significant structural conformational changes occurring for antibody dissolved in PBS in comparison to that for antibody dissolved in RTIL. The degradation in α-helix and β-sheet structures is prominent starting only at T_{96} hours for antibody dissolved in RTIL, while the degradation has occurred well before T_{48} hours for antibody dissolved in PBS.

We evaluated the temporal stability of α-IL-6 antibody over time in 50% RTIL. Figure S2 presents the R_h and zeta potential at T_0, T_{24}, T_{48}, and T_{96} hours. The size remained constant at 1± 0.04 nm with time indicating that no aggregates are formed and the zeta potential varied from 27.9–40.2 mV. A slight increase in zeta potential from 27.9 mV at T_0 hour to 40.1 mV at T_{24} hour could be attributed to settling time of the protein as also seen in the ATR-IR results where the Amide I peak at 1665 cm^{-1} becomes prominent on the post 24 hour spectra. Although the zeta potential drops slightly to 34.8 mV at T_{96} hours no aggregates were formed confirming the stability of the protein antibody for over 96 hours in 50% RTIL solution and correlates with ATR-IR results.

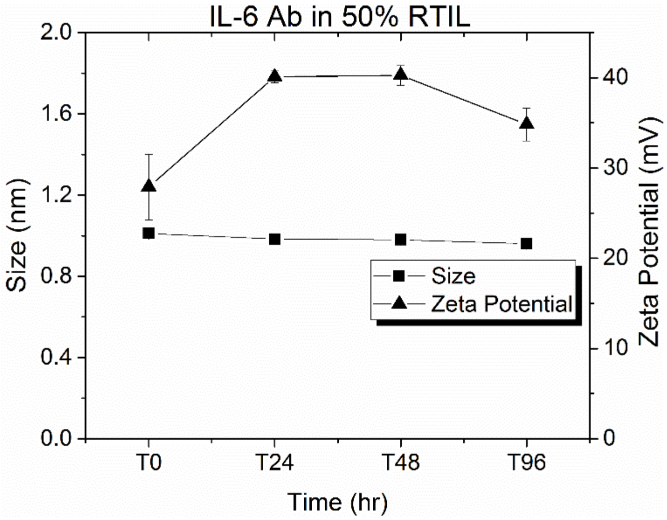

Figure S2. DLS and zeta potential measurements of temporal stability of α-IL-6 antibody

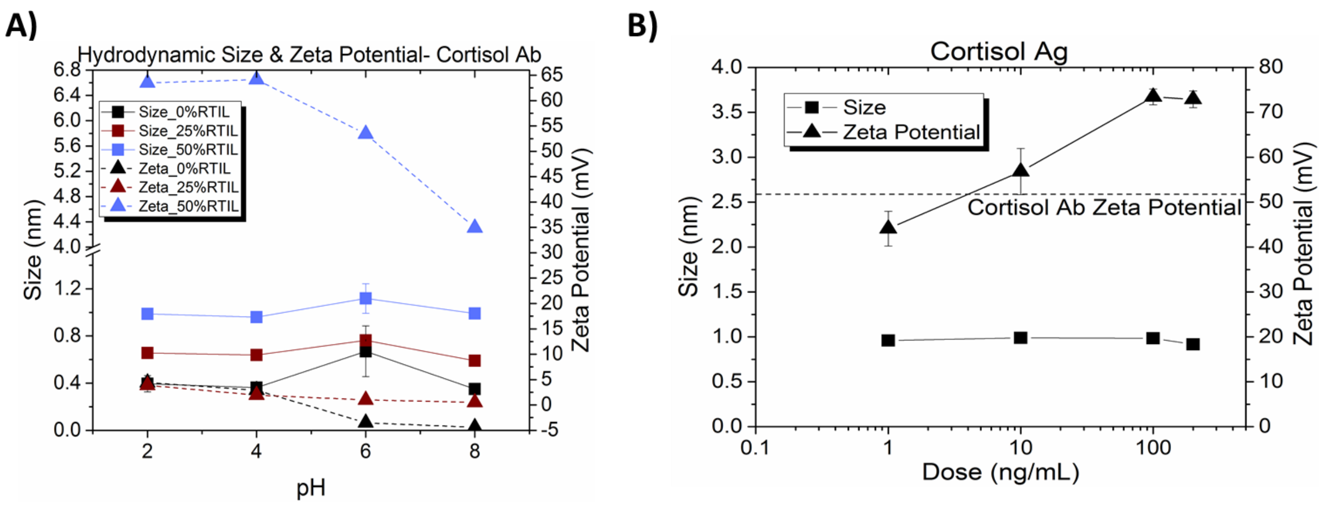

Figure S3. DLS and zeta potential measurements. A) α-Cortisol antibody spiked in synthetic sweat (SS) of varying pH and RTIL ratios; C) cortisol antibody-antigen interactions in 50% RTIL in SS solutions. The dotted line in B) represent the zero dose antigen zeta potential value for the respective proteins.

From Fig. S3A, the R_h of α-cortisol varies at 0% RTIL across pH from 0.4 nm to 0.6 nm. However, it remains constant across pH at 0.7 nm and 1.1 nm for RTIL volumetric ratio of 25% and 50% respectively. From zeta potential graph in Fig. S3A, for 0% RTIL solution the zeta potential varies from 3 mV to -5 mV across pH range from 2 to 8, with the crossover occurring at pH 5. The zeta potential differs from 3 mV to 0.5 mV across pH range from 2 to 8 in 25% RTIL, and at 50% RTIL the zeta potential varies from 65 mV to 34 mV across pH range from 2 to 8 respectively. The conclusions

from these results indicate that for α-cortisol antibody, the zeta potential varies with the pH of SS. Figure S3B represent the cortisol antibody-antigen interactions in 50% RTIL in SS solutions for varying doses of antigen. The R_h of molecules remains constant at 1± 0.2 nm and zeta potentials increases from 40 mV to 70 mV with increasing antigen dose concentrations.

Combinatorial cortisol detection and specificity in human sweat using EIS

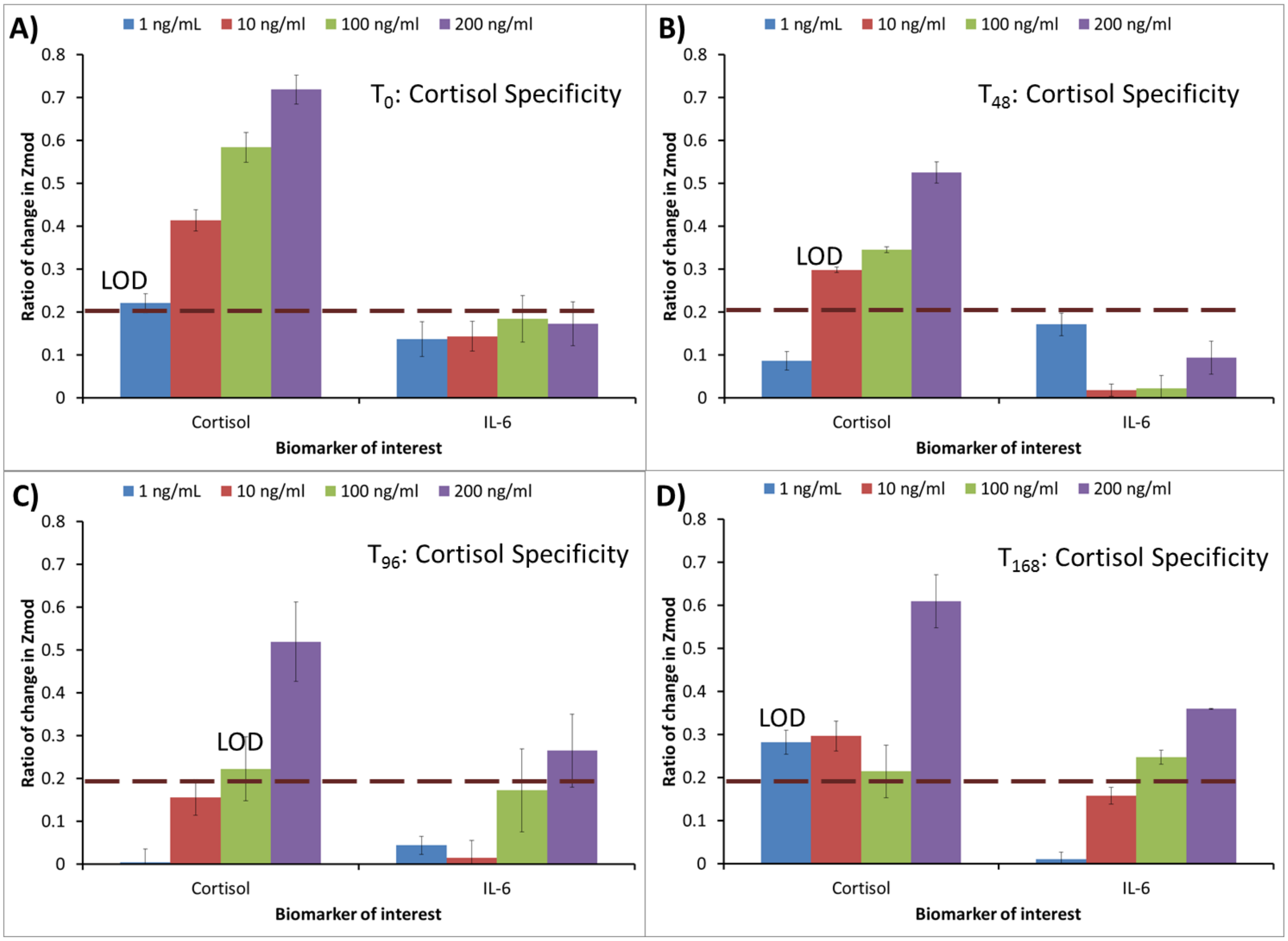

Figure S4. Cortisol antibody specificity towards cortisol antigen and non-specific interactions with IL-6 antigen for time intervals of antibody storage of T_0 to T_{168} hours.

We performed experiments using cortisol antibody for cortisol detection as described in the Materials and Methods section, in order to understand the versatility of developed sensor using RTIL bioimmunoassay functionalized semiconducting ZnO thin films deposited on nanoporous, flexible

polymer membranes in combinatorial detection performance and protein stability. α-cortisol antibody diluted in BMIM[BF_4] was incubated after DSP linker functionalization on the ZnO surfaces. This was followed by dispensing 3–5 μL volume of human sweat to establish the zero dose or baseline EIS measurement. Post baseline measurement, cortisol dilutions in human sweat of 1 ng/mL to 200 ng/mL concentrations were serially dispensed and measured using EIS. Specificity of assay was established using IL-6 dilutions in human sweat from 1ng/mL to 200 ng/mL on the α-cortisol immobilized sensor surfaces. In case of temporal studies, antibody immobilization was done on separate sensors at room temperature and was stored in 4°C for 24, 48, 96, and 168 hours before EIS measurements were taken.

The clinical range of cortisol in human sweat is established to be 8–140 ng/mL and therefore, cortisol concentrations from 1–200 ng/mL were spiked in human sweat and tested on cortisol antibody functionalized sensor surface. The specificity of this assay was cross-verified by evaluating its response to non-specific molecule IL-6 using EIS. The results of these experiments with the ratio of change in Z_{mod} are displayed in Figure S4. The signal threshold SST ratio in these experiments was 0.208.

The range in ratio of change in Z_{mod} is from 0.2 to 0.7 for cortisol concentration ranging from 1 ng/mL to 200 ng/mL for T_0 with LOD at 1 ng/mL. The range in ratio of change in Z_{mod} decreases from 0.1 to 0.52 for cortisol concentration ranging from 1 ng/mL to 200 ng/mL for T_{48} with LOD at 10 ng/mL, which meets lower concentration limit established for the clinical range of cortisol in human sweat. At T_{96} and T_{168} hours, the response of the cortisol antibody towards cortisol detection is diminished for lower concentrations causing the LOD to be at 200 ng/mL. Thus there is a linear calibration response for cortisol detection until T_{48} hours. The signal for non-specific molecule IL-6 is observed to be well below SST of 0.2 at T_0 and T_{48} hour time intervals for all concentrations. At T_{96} and T_{168} hours, only the higher IL-6 concentration 200ng/mL is above the SST at ratio of 0.25 and 0.4 respectively. We hypothesize the relative differences in the stability of the molecules IL-6 and Cortisol

i.e. T_{96} and T_{48} on the developed sensor is likely due to the differences in EDL characteristics of these molecules in the BMIM[BF_4] RTIL.

Table S1: Comparison of detection of biomolecules in various buffer using electrochemical detection techniques

Analyte Detected	Buffer Medium	Physiological range	Detection Method	Sensitivity / LOD* *all values reported at T_0 unless specified	Reference
Lactate	Human Sweat	≤ 2.22mg/mL	Amperometry	8.9 µg/mL	[2]
Cortisol	Human Sweat	10 – 200 ng/mL	EIS	1 ng/mL at T_0<br>10 ng/mL at T_{48}	[4],*This Work*
Cortisol	Human Sweat		Optical	8.16 ng/mL	[5]
Interleukin-6	Serum	7 – 16 pg/mL	Amperometry	1.37 pg/mL	[6]
Interleukin-6	Artificial Sweat		EIS	0.02 pg/mL	[7]
Interleukin-6	Human Sweat		EIS	0.2 pg/mL at T_0<br>5 pg/mL at T_{48}	*This Work*

References

[1] J. Buijs, W. Norde, J.W.T. Lichtenbelt. *Langmuir*, **12** (1996) 1605-1613.

[2] Jia, W. et al. Electrochemical tattoo biosensors for real-time noninvasive lactate monitoring in human perspiration. *Analytical Chemistry*, **85** (2013) 6553-6560.

[3] Guinovart, T., Bandodkar, A.J., Windmiller, J.R., Andrade, F.J. &Wang, J. A potentiometric tattoo sensor for monitoring ammonium in sweat. *Analyst*, **138** (2013) 7031.

[4] Munje, R. D., Muthukumar, S., Selvam, A.P. & Prasad, S. Flexible nanoporous tunable electrical double layer biosensors for sweat diagnostics. *Nature Scientific Reports*, **5** (2015).

[5] Russell, E., Koren G., Rieder M.J., Van Uum S.H. The detection of cortisol in human sweat: implications for measurement of cortisol in hair. *Therapeutic Drug Monitoring*, **36 (**2013).

[6] Chen et al. Label-free electronic detection of interleukin-6 using horizontally aligned carbon nanotubes. *Materials and Design*, **90** (2016) 852-857.

[7] Selva Kumar, L.S et al. Label free nano-aptasensor for interleukin-6 in protein-dilute bio fluids such as sweat. *Analytical Methods*, **8** (2016) 3440.
